# Dr. Joseph Jones

**Published:** 1896-03

**Authors:** 


### Obituary.

---

Dr. Joseph Jones, one of the most noted southern physicians and scientists, died February 18, 1896, at his home in New Orleans. He was born in Georgia in 1833, was graduated at Princeton in 1853 and at the medical department of the University of Pennsylvania in 1855. He was professor of chemistry in the Medical College of Savannah in 1856, of natural philosophy in the University of Georgia in 1858, and of chemistry in the Medical College of Georgia from 1859 to 1865. From 1862 to 1865 he served as surgeon in the Confederate Army. From 1866 to 1868 he was professor of medicine in the University of Nashville, and from 1869 up to a few years ago was professor of chemistry and clinical medicine in Tulane University.

He was president of the Louisiana State Board of Health from 1880 to 1884, and while serving in this capacity was successful in excluding yellow fever from the Valley of the Mississippi. Dr. Jones devoted his life to the investigation of the causes and prevention of disease in civil and military hospitals, and was a prolific writer on the subject of his experiments. He was the author of a score of published works, besides innumerable pamphlets and addresses. In addition to his medical works he published a book on the agricultural resources of Georgia and several on Southern Indians. He married a daughter of Lieutenant-General (Bishop) Leonidas Polk, C. S. Army.

